# *m*-Polar fuzzy based image enhancement using exposure fusion

**DOI:** 10.3389/frai.2026.1940368

**Published:** 2026-10-30

**Authors:** Mahesh Kumar C. V., Saraswathi D.

**Affiliations:** 1 Department of Mathematics, School of Advanced Sciences, Vellore Institute of Technology, Chennai, India; 2 Centre for Human Movement Analytics, School of Computer Science and Engineering, Vellore Institute of Technology, Chennai, India

**Keywords:** exposure fusion, fuzzy set, image enhancement, image fusion, low light enhancement

## Abstract

Low light image enhancement is a fundamental pre-processing step in numerous image-processing applications such as medical image analysis, underwater imaging and so on. Many conventional low-light image enhancement methods are typically designed for uniformly dark inputs and tend to over-brighten or distort normally exposed regions in mixed-illumination scene. Since mixed-illumination scenes contain region with differing exposure levels, a single enhancement rule is not adequate; multiple rules must instead be applied jointly within a single image. To address this, we propose a enhancement framework based on *m*-polar fuzzy set theory where each polar corresponds to a distinct enhancement rule. The input image is transformed into *m* polar images using parametric fuzzy complement functions, and these polar images are then fused through a multi-scale Laplacian pyramid strategy, with pixel-wise weight maps constructed from contrast, saturation, and well-exposedness. Negative-γ polarities suppress over-brightening in well-lit regions, while positive-γ polarities recover detail in dark areas, enabling selective enhancement without distorting normally exposed content. Experimental evaluation on the TM-DIED and ExDark benchmarks against nine state-of-the-art methods demonstrates that the proposed approach achieves competitive performance across structural similarity index (SSIM), Absolute Mean Brightness Error (AMBE), Lightness Order Error (LOE), Lightness Order Measure (LOM), Structure Measure Operator (SMO), Blind/Referenceless Image Spatial Quality Evaluator (BRISQUE), Entropy, Perception based Image Quality Evaluator (PIQE), Fitness, and Natural Image Quality Evaluator (NIQE) metrics. The source code is available at https://github.com/maheshkumar-cv/m-polar-fuzzy-low-light-image-enhancement.

## 1 Introduction

Image enhancement is widely used pre-processing operation in applications, including medical image analysis (Thien et al., 2025), plant and animal disease (Uferah et al., 2022), autonomous navigation (Guofa et al., 2021), face recognition (Yu-Hsuan and Homer, 2022), and underwater imaging (Weidong et al., 2022). It refers to the modification of image representation to improve perceptual quality in terms of brightness, contrast, noise suppression, and related characteristics for subsequent processing tasks. Existing enhancement techniques can be broadly categorized into histogram-based (Karel, 1994), fuzzy-based, geometric function theory-based (Rahman et al., 2025) and deep learning-based methods. Histogram-based methods (BECE—Rahman and Shimamura, 2026a, RSFHE—Rahman and Shimamura, 2026b) are computationally simple, but they are limited by their reliance on histogram adjustment alone, which may not produce visually satisfactory results for low light image. Rahman and Shimamura (2025) proposed Dualistic fuzzy histogram equalization (DFHE) that computes a fuzzy image histogram (FIH) and partitions the image into two sub-histograms at a computed partitioning level, equalizing each independently to better preserve mean brightness and entropy. From Figure 1 it can be clearly seen that the histogram based methods does not produce visually satisfactory results for low light image enhancement. Deep learning-based enhancement methods are generally classified into supervised and unsupervised approaches. Supervised methods require ground-truth images, which are often unavailable in practical scenarios, which makes them poorly suited for real-time applications. Although unsupervised methods eliminate the need for ground truth, they usually involve multiple processing layers, which increases computational cost and makes them less suitable for real-time use. For example EnlightenGan (Jiang et al., 2021) adopts a U-Net generator guided by attention map derived from the image's illumination, paired with global and local discriminators for region-level enhancement quality. Such multi-stage, multi-layer designs substantially increase inference time. This limitation becomes more significant when enhancement is used as a pre-processing step in low-light face detection or recognition, since the additional processing burden increases overall time complexity. Moreover, such methods often require expensive GPU resources to store and process model parameters. As a result, recent research has increasingly focused on fuzzy-based methods, where the lack of illumination is treated as a fuzzy condition and fuzzy operations are used to restore the illumination characteristics of the image, requiring only *O*(*mn*) time for an image of size *m*×*n* (Maheshkumar et al., 2025a).

**Figure 1:**
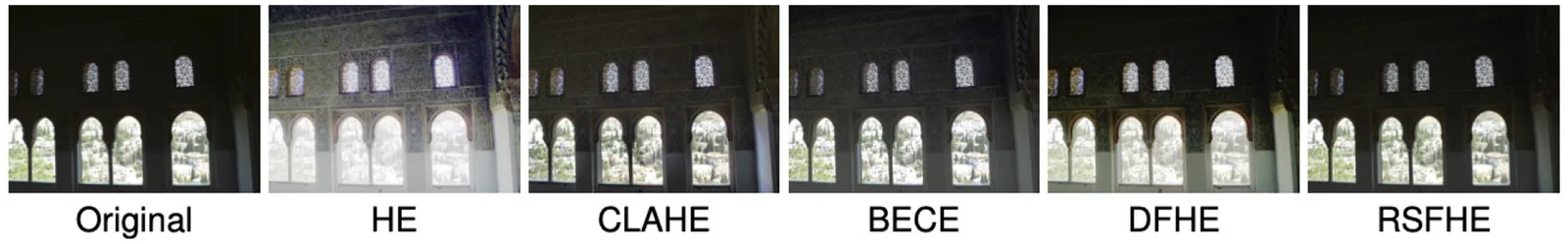
Comparison of equalization based methods (image adapted with permission from The Most Difficult Image Enhancement Dataset [TM-DIED], Vasileios, V. [2025], https://sites.google.com/site/vonikakis/datasets/tm-died).

A popular approach for constructing a fuzzy image is by using a complement function (Hauβecker and Tizhoosh, 2000; Maheshkumar et al., 2025a) that contains a parameter γ, which controls the degree of enhancement applied to a given image. In the literature, the value of γ has been determined using manual tuning, naive methods, search algorithms, and optimization techniques. Since fuzzy enhancement is a global operator, a single γ value is applied to all pixels. However, this assumption is not suitable in practice because different pixels may require different levels of enhancement. Therefore, in this article, we use an *m*-polar fuzzy set to generate *m* differently exposed images from a given image, which are then fused and defuzzified to obtain the final enhanced image. There are several ways to fuse these images, among which pyramid-based and deep learning-based methods are highly effective. As noted earlier, deep learning-based methods are not reliable for real-time applications. In contrast, since a low-light image's varying illumination across regions requires pixel-level enhancement rather than global enhancement and a pyramid-based fusion performs pixel-level fusion, a pyramid-based fusion suits fuzzy-based processing well.

Pyramid representations such as Gaussian and Laplacian (Mertens et al., 2007), Wavelet (Hsu and Jian, 2024), Morphological (Toet, 1989) and so on, provide an efficient multiscale framework for image analysis and processing. Among the various pyramid structures, the Gaussian pyramid and the Laplacian pyramid are the most widely used. These representations are extensively employed in image compression, enhancement, blending, and edge-preserving filtering. The Gaussian pyramid is a multilevel representation obtained by repeatedly applying a Gaussian low-pass filter followed by downsampling. At each successive level, the image is represented at a reduced spatial resolution. In the standard implementation, the input image is convolved with a Gaussian kernel and then subsampled by a factor of two. This procedure is iterated to produce a hierarchical representation that captures image content from fine to coarse scales. The Laplacian pyramid is derived from the Gaussian pyramid. Each level is formed by subtracting the expanded version of the next coarser Gaussian level from the current level. Consequently, the Laplacian pyramid provides a band-pass representation of the image, isolating details at different scales and preserving local image structures effectively.

In short, the motivation of this study is to overcome the limitation of existing fuzzy-based enhancement methods, which apply a single parameter to enhance the image and are therefore unable to adequately handle images with multiple illumination regions, calling for a method that adapts to varying illumination at the pixel level. To achieve this, in this paper, we propose an *m*−polar fuzzy set based image enhancement method using exposure fusion, which constructs multiple fuzzy images (polars) of the input image at varying parameter values and fuses them using a pyramid-based fusion strategy, thereby achieving adaptive enhancement while preserving brightness and minimizing artifacts across images with multiple illumination regions.

The main contributions of this article are summarized as follows:

- An *m*-polar fuzzy is utilized to generate multiple exposure representations of the input image.
- Pyramid-based multiple exposure fusion is applied to the generated *m*-polar images to combine complementary information.
- A defuzzification function is employed to obtain the final enhanced image from the fused result.

## 2 Related works

### 2.1 Multi exposure image fusion

Burt and Kolczynski (1993) was among the first to investigate multi-exposure fusion (MEF) by proposing a gradient pyramid model based on directional filtering. Later, Mertens et al. (2007) introduced a pixel-wise exposure fusion method within a multiresolution framework using Gaussian and Laplacian pyramids. Their work attracted significant attention and motivated many subsequent improvements aimed at enhancing fusion quality and computational efficiency. For example, Song et al. (2011) proposed a probabilistic exposure fusion model that maintains luminance consistency while reducing gradient reversal artifacts in image brightness. Shen et al. (2014) further refined the Laplacian pyramid approach by incorporating a boosting strategy to separate base and detail layers, together with a hybrid weighting scheme driven by both the exposure level of individual images and their relative differences across multiple exposures. Li et al. (2017) presented a detail-enhanced fusion framework based on a weighted guided image filter, where a weighted structure tensor was introduced to guide detail extraction. This allowed the amount of fine-detail enhancement to be adjusted according to user preference. Wang et al. (2019) proposed a low-complexity detail-enhanced fusion algorithm in the YUV color space, using a simple vector-field construction method to reduce detail degradation during fusion. Jishnu and Vishnukumar (2023) presented an MEF method designed for a limited number of severely exposed static input images. Their approach first generated an intermediate exposure stack through exposure correction and then applied pyramid-based fusion (Mertens et al., 2007) method. More recently, Akbulut and Aslantas (2026) proposed a block-based MEF model optimized using a genetic algorithm.

Deep learning-based MEF methods are generally categorized into supervised learning and unsupervised learning approaches. Yu et al. (2020) proposed a CNN-based multimodal image fusion framework that uses two convolutional layers to extract low-level features from multiple input images and combines them through alternative fusion strategies. The fused features are then passed through another pair of convolutional layers to generate the final fused image. Tang and He (2025) employed a focal transformer to emphasize salient regions and model long-range relationships among multiple exposures, while adversarial learning was used to train the network with ground-truth guidance. Since supervised methods are not well-suited to real-time imaging scenarios, attention has shifted toward unsupervised approaches. Ma et al. (2015) introduced the no-reference quality metric MEF-SSIM, which is highly useful as a loss function in unsupervised learning. Li and Zhang (2018) extracted features from a pretrained CNN originally trained for other tasks and used them to compute weight maps, making the method suitable for dynamic scenes. Bai et al. (2025) decomposed multiple exposures into illumination components with a shared reflectance term. Their reflectance was learned using a bidirectional loss, and a controllable exposure fusion criterion was introduced to support global exposure adjustment while preserving contrast, thereby overcoming the limitation of fixed exposure settings.

### 2.2 Fuzzy image processing

Although fuzzy sets provide a well-established mechanism for modeling uncertainty via membership functions, leveraging these functions to extract richer image information has historically proven difficult. To overcome this shortcoming, Chaira (2014b) proposed a novel membership function derived from the Hamacher t-conorm operator, which facilitated medical image enhancement within a type-2 fuzzy set framework. In a related effort, Ghosh and Ghosh (2022) presented an innovative mammogram enhancement scheme by formulating a hyperbolic regularization-based adaptive intuitionistic fuzzy divergence measure. Their approach employed Werner's AND-OR operators to construct a reformulated membership function, thereby enabling more resilient and adaptive enhancement across diverse imaging scenarios.

A well-known drawback of intuitionistic fuzzy image (IFI) construction is that the resulting gray-level distribution tends to shift toward higher intensity values, leading to diminished contrast and loss of visual fidelity. To mitigate this issue, histogram-based approaches have been incorporated into IFI-based frameworks, giving rise to a series of improved low-light image enhancement (LLIE) models. Jebadass and Balasubramaniam (2022b) put forward an LLIE model grounded in Yager's intuitionistic fuzzy generator (IFG), utilizing histogram equalization (HE) to improve image quality. A subsequent extension of the work Jebadass and Balasubramaniam (2022a) adopted an interval-valued intuitionistic fuzzy set (IVIFS) framework for LLIE, substituting HE with the contrast-limited adaptive histogram equalization (CLAHE) technique to control the over-enhancement and noise amplification. Continuing in this direction, Jebadass and Balasubramaniam (2024) constructed an additional LLIE model based on the IFG introduced in Chaira (2020), further consolidating the role of IFG in enhancement pipelines. Concurrently, Chithra et al. (2024) put forward a new IFG specifically formulated for LLIE, also leveraging CLAHE to address low-contrast conditions. Extending this line of inquiry, Chinnappan and Sundaram (2024) devised a distinct IFG for LLIE with a focus on low-light video sequences, incorporating HE as a component of the overall enhancement pipeline. More recently, Ragavendirane and Dhanasekar (2025) proposed an IFG-based LLIE strategy that integrates a HE-driven Retinex decomposition into the enhancement process. In a parallel development, Selvam and Sundaram (2025) formulated an IVIFS-based LLIE model by generalizing the IFG from Chithra et al. (2024), incorporating the structural similarity index (SSIM) as a reference-guided quality metric to steer the enhancement toward ground-truth alignment. It is worth noting that this method is applicable only to images for which a ground-truth reference is available, as SSIM computation inherently requires a ground-truth image to guide the enhancement process, thereby limiting its use in real-time or ground-truth-unavailable scenarios.

Given the proliferation of IFG-based methods in the image enhancement literature, there has been a growing impetus to unify these under a common generalized framework. Toward this goal, Maheshkumar et al. (2025a) introduced a generalization of Sugeno's IFG, wherein numerous previously proposed IFGs emerge as special cases. Their formulation relies on a continuous function *h*(γ) embedded within the IFG definition. Alongside this theoretical contribution, they developed a dedicated search procedure, termed BestIFI, to optimally tune the enhancement parameters, and employed it within a complete LLIE model. This search strategy was subsequently extended to the IVIFS setting in Maheshkumar et al. (2025b). Specifically, by assigning *h*(γ) to *e*^γ^−1, γ+1, (γ−1)^2^, 1/γ^2^−1, 3γ^2^+4γ+1, (γ+1)^2^, (γ+1)^3^, and γ*e*^γ+1^, the framework recovers the IFGs originally presented in Chaira (2014a, 2020, 2021), Haribabu and Guruviah (2023), Chinnappan and Sundaram (2024), Chithra et al. (2024), Ragavendirane and Dhanasekar (2025), and Maruturi and Velmathi (2025), respectively. Also, Yogambaram et al. (2026) uses reptile search algorithm in the IVIFS to optimize the parameters. Beyond intuitionistic fuzzy sets, extended fuzzy paradigms including Pythagorean, Fermatean, and *q*-rung orthopair fuzzy sets, construct fuzzy images in a manner analogous to IFIs, with the hesitancy component derived according to each framework's defining constraints. In this context, Chaira and Sarkar (2024) introduced a Pythagorean fuzzy generator for constructing Pythagorean fuzzy images and applied a fuzzy intensification operator to enhance low-contrast mammogram images. Additionally, a bipolar fuzzy set-based enhancement model was proposed by Mahesh Kumar et al. (2026); however, the corresponding fuzzy image representation incurs notable detail loss, and the reliance on CLAHE as a supplementary enhancer means the model inherits the known limitations associated with that method.

## 3 Methodology

This section presents the proposed framework for low-light image enhancement. We begin by constructing an *m*-polar fuzzy representation of the input image, wherein each polarity captures a distinct aspect of the pixel intensity distribution. The resulting polar images are subsequently fused using a multi-scale pyramid-based exposure fusion strategy (Mertens et al., 2007), and the final enhanced image is recovered through defuzzification. The flowchart of the proposed method is given in Figure 2. The pseudocode for the proposed model is given in Algorithm 1.

**Figure 2:**
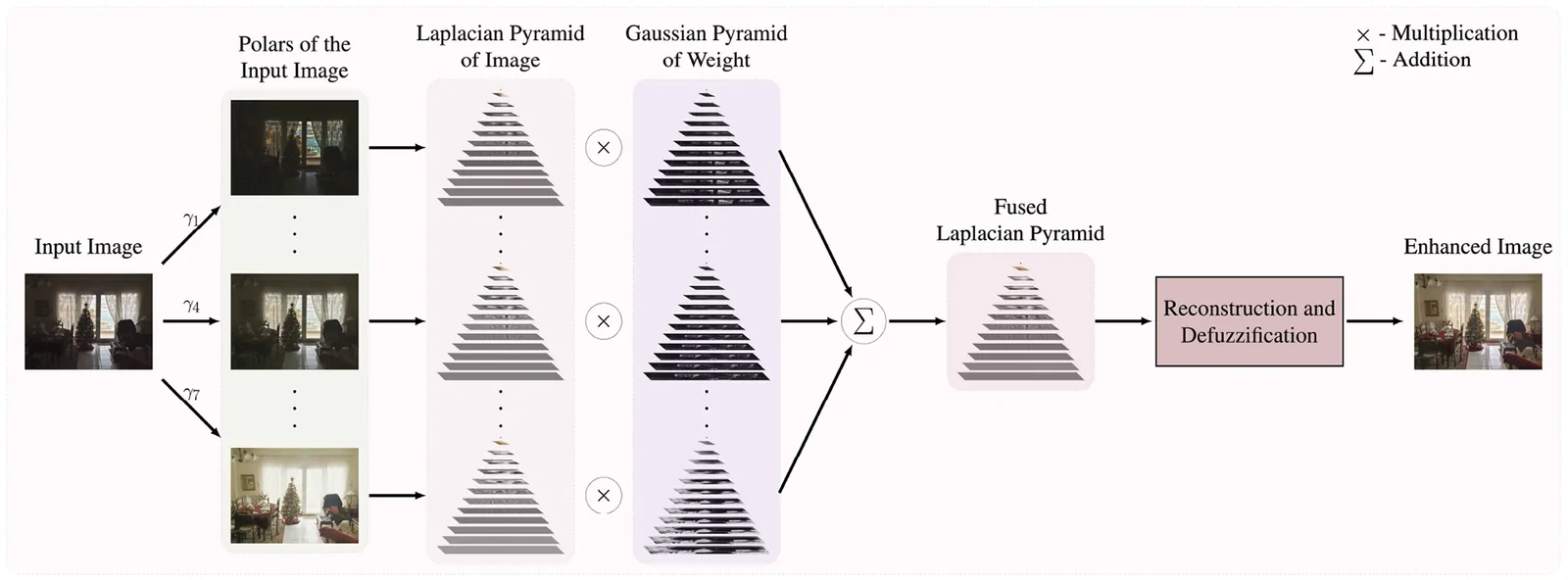
Flowchart of the proposed method. The input image is first transformed into *m* polar images via parametric fuzzy complement functions (image adapted with permission from The Most Difficult Image Enhancement Dataset [TM-DIED], Vasileios, V. [2025], https://sites.google.com/site/vonikakis/datasets/tm-died). The Laplacian pyramid of each polar image and the Gaussian pyramid of the corresponding weight map, computed from contrast, saturation, and well-exposedness, are then multiplied level-wise and summed across all polarities to yield a fused Laplacian pyramid. The fused pyramid is reconstructed into a single image and subsequently defuzzified to produce the final enhanced output.

Algorithm 1. m-polar fuzzy exposure fusion. 
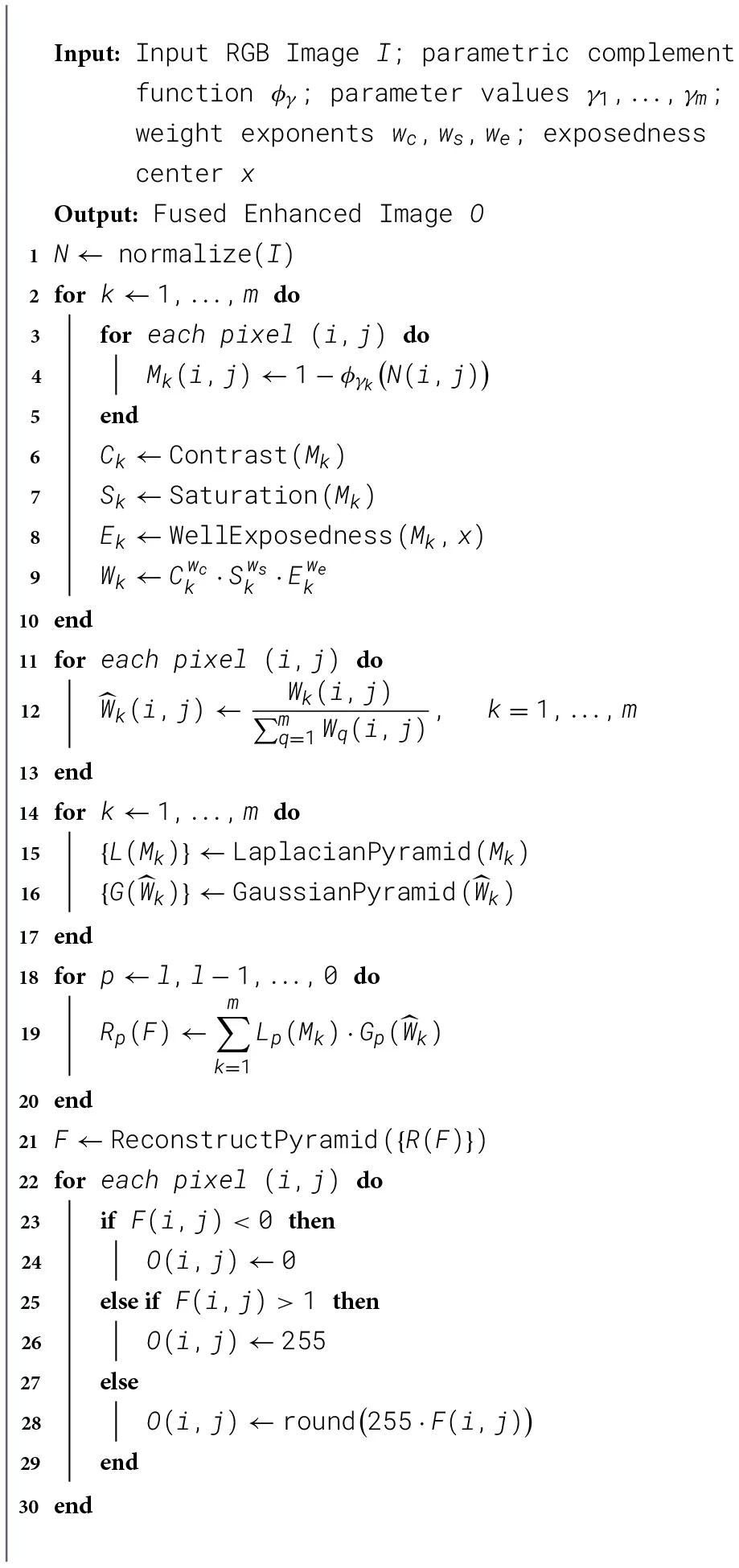

### 3.1 Construction of *m*-polar fuzzy sets and polar images

#### 3.1.1 Fuzzy sets and *m*-polar fuzzy sets

A classical fuzzy set $\tilde{F}$ over a universe *A* is expressed as

$$\tilde{F}=\left\{\left(a,\mu_{\tilde{F}}\left(a\right)\right)|a\in A\right\},$$

where the membership function $\mu_{\tilde{F}}:A\to \left[0,1\right]$ assigns to each element *a*∈*A* a degree indicating the extent to which *a* belongs to $\tilde{F}$.

The notion of an *m*-polar fuzzy set generalizes this by allowing each element to carry *m* independent membership degrees simultaneously. Formally, an *m*-polar fuzzy set on a domain *X* is a mapping $A:X\to {\left[0,1\right]}^{m}$, so that every *x*∈*X* is associated with an ordered *m*-tuple (*μ*_1_(*x*), *μ*_2_(*x*), …, *μ*_*m*_(*x*)), where each component *μ*_*i*_(*x*) represents the degree of membership with respect to the *i*-th polarity. This multi-polarity structure enables richer characterization of pixel intensities than a single membership value can provide.

#### 3.1.2 Fuzzy complement and the first characterization theorem

To generate the individual membership functions μ_1_, …, μ_*m*_, we employ involutive fuzzy complement functions. The theoretical foundation for constructing such complements is given by the following classical result.

**Theorem 1** (First Characterization Theorem). *A function ϕ*:[0, 1] → [0, 1] *is an involutive fuzzy complement if and only if there exists a continuous, strictly increasing function f*:[0, 1] → ℝ *with f*(0) = 0 *such that, for all a*∈[0, 1],

$$\phi \left(a\right)=f^{-1}\left(f\left(1\right)-f\left(a\right)\right).$$

Leveraging this theorem, we adopt the following parametric complement function by Maheshkumar et al. (2025a):

$$\begin{array}{l} f\left(a\right)=\frac{1}{h\left(\gamma \right)}\log\left(1+h\left(\gamma \right)a\right),\;f^{-1}\left(a\right)=\frac{e^{h\left(\gamma \right)a}-1}{h\left(\gamma \right)} \end{array}$$

with *h*(γ) = tan(γ), so

$$\begin{array}{l} \phi_{\gamma }\left(x\right)=\frac{1-x}{1+\tan\left(\gamma \right)x},\;\gamma \in \left[0,pi/2\right), \end{array}$$ (1)

Different choices of γ yield distinct mappings from pixel intensity to membership degree, thereby producing polar images with varied contrast and brightness characteristics.

#### 3.1.3 Constructing polar images

For *i* = 1, 2, …, *m*, the *i*-th membership function is defined as the complement of the normalized pixel intensity:

$$\begin{array}{l} \mu_{i}\left(x\right)=1-\phi_{\gamma_{i}}\left(x\right), \end{array}$$ (2)

where γ_*i*_ is the parameter associated with the *i*-th polarity. The subtraction from unity ensures that μ_*i*_ acts as an enhancement operator: dark pixels (low *x*) are mapped to higher membership values, which brightens the corresponding polar image.

The *m*-polar fuzzy set $\tilde{W}$ of the input is then written as

$$\tilde{W}=\left\{\left(x,\mu_{1}\left(x\right),\mu_{2}\left(x\right),\ldots ,\mu_{m}\left(x\right)\right)|x\in X\right\}.$$

Let *N* denote the pixel-wise normalized version of the input image *I* (i.e., *N*_*mn*_∈[0, 1] for every pixel position (*m, n*)). The pixel value at position (*m, n*) in the *i*-th polar image *P*_*i*_ is obtained by evaluating the corresponding membership function:

$${\left(P_{i}\right)}_{mn}=\mu_{i}\left(N_{mn}\right).$$

Thus, the *m* polar images *M*_1_, *M*_2_, …, *M*_*m*_ constitute a set of different representations of *I*, each emphasizing different tonal ranges according to its governing parameter γ_*i*_. These images are subsequently combined through the pyramid-based exposure fusion described in Section 3.4.

### 3.2 Pyramid construction and reconstruction

Multi-scale pyramid decomposition is used to fuse the polar images while preserving both coarse structure and fine detail. We employ two complementary pyramids: the Gaussian pyramid, which represents progressively smoothed and sub-sampled versions of an image, and the Laplacian pyramid, which encodes the band-pass residuals between consecutive Gaussian levels. The pyramid construction and image reconstruction from pyramid follows Burt and Kolczynski (1993).

#### 3.2.1 Gaussian pyramid

Let *G*_0_ = *I* be the original image. Each subsequent level *G*_*i*_ of the Gaussian pyramid is produced by first blurring *G*_*i*−1_ with a Gaussian kernel to suppress high-frequency content, and then downsampling the result by a factor of two:

$$\begin{array}{l} G_{i}=\mathrm{Downsample}\left(\mathrm{GaussianBlur}\left(G_{i-1}\right)\right). \end{array}$$ (3)

Pre-blurring before downsampling is essential to avoid aliasing artifacts. The resulting pyramid {*G*_0_, *G*_1_, …, *G*_*l*_} provides a coarse-to-fine representation of the image. The number of pyramid levels is determined by the smaller image dimension. The maximum depth *l* = ⌊log_2_min(*r, c*)⌋, i.e., the largest *l* such that 2^*l*^ ≤ min(*r, c*). Each level is obtained by Gaussian smoothing followed by subsampling by a factor of two, so that *G*_*i*_ has size ⌈*r*/2^*i*^⌉ × ⌈*c*/2^*i*^⌉ (odd dimensions are rounded up). The pseudocode for the Gaussian pyramid is given in Algorithm 2.

Algorithm 2. GaussianPyramid(*I*). 
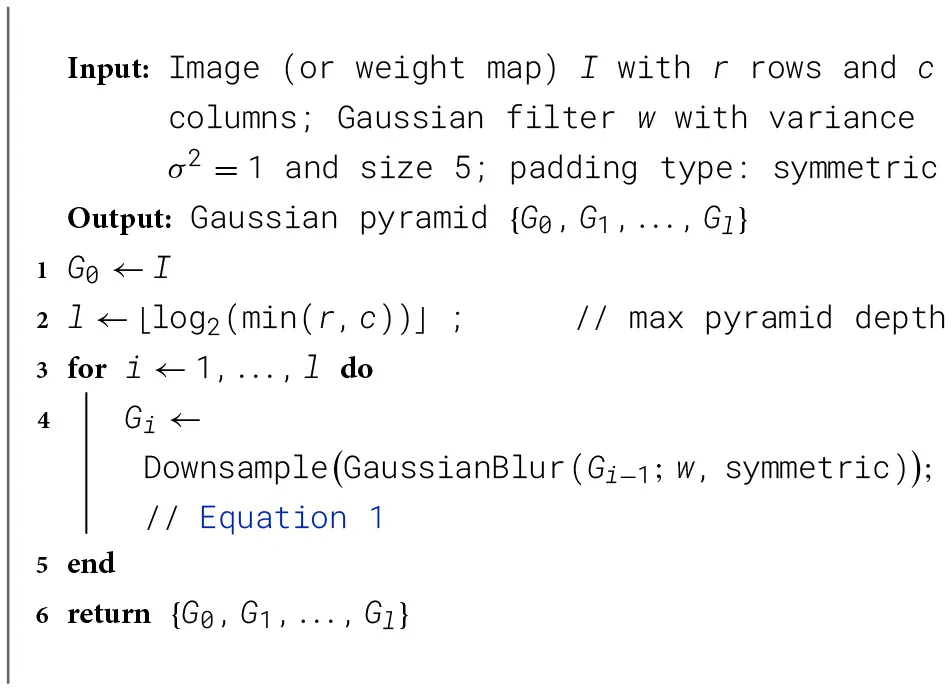

#### 3.2.2 Laplacian pyramid

The Laplacian pyramid captures the detail information discarded at each level of the Gaussian pyramid. Given two consecutive Gaussian levels *G*_*i*_ and *G*_*i*+1_, the *i*-th Laplacian level is:

$$\begin{array}{l} L_{i}=G_{i}-\mathrm{Upsample}\left(G_{i+1}\right), \end{array}$$ (4)

where the upsampling operation expands *G*_*i*+1_ to the spatial resolution of *G*_*i*_ via bilinear interpolation followed by Gaussian filtering to suppress interpolation artifacts, with appropriate cropping to ensure size consistency. Since, *G*_*l*+1_ is not available *L*_*l*_ = *G*_*l*_. The pseudocode for the Gaussian pyramid is given in Algorithm 3.

Algorithm 3. LaplacianPyramid(*I*). 
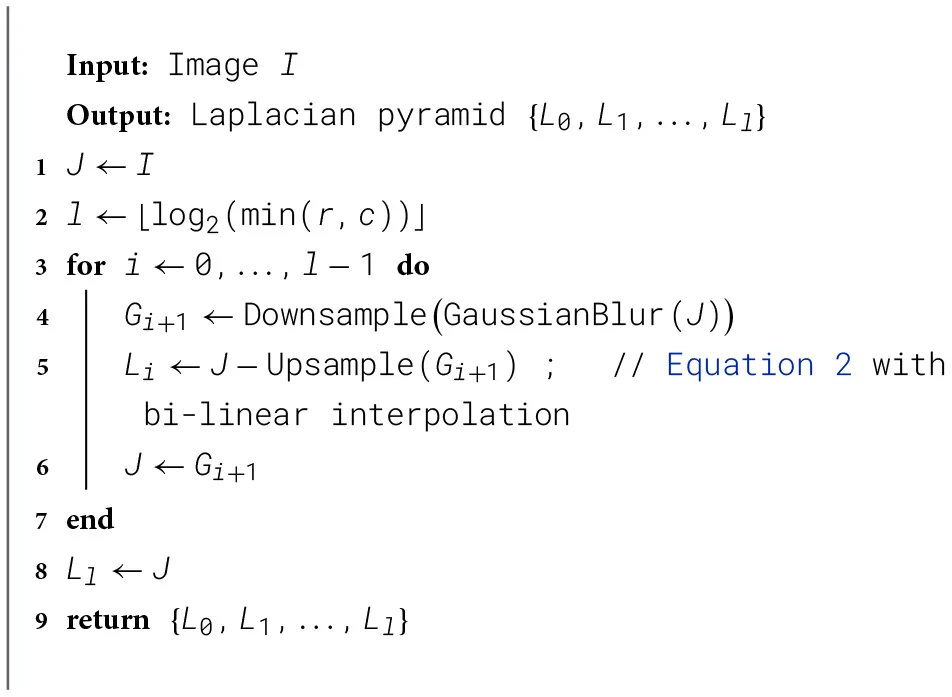

#### 3.2.3 Pyramid reconstruction

The full-resolution image is recovered from the Laplacian pyramid through a bottom-up reconstruction process. Starting from the coarsest level, where *R*_*l*_ = *L*_*l*_, each finer level is reconstructed recursively as:

$$\begin{array}{l} R_{i}=L_{i}+\mathrm{Upsample}\left(R_{i+1}\right). \end{array}$$ (5)

Repeating this until *i* = 0 yields the reconstructed image *R*_0_. The pseudocode for the image reconstruction from pyramid is given in Algorithm 4.

Algorithm 4. ReconstructPyramid({*L*_0_, *L*_1_, …, *L*_*l*_}). 
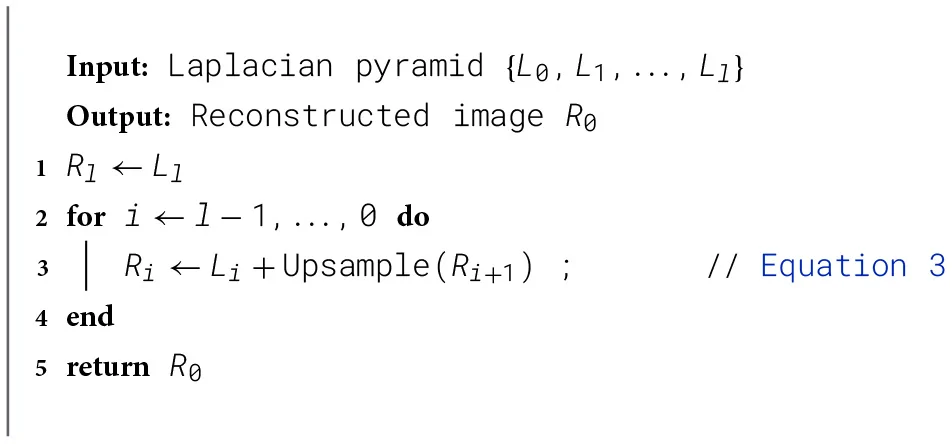

### 3.3 Construction of the weight map

For each polar image, a pixel-wise quality weight map is constructed by combining three perceptual measures: contrast, saturation, and well-exposedness. These measures capture complementary aspects of image quality, and their product forms a scalar weight at every pixel that guides the subsequent fusion step.

1. Contrast: Contrast measures the local edge strength, ensuring that sharper regions receive higher fusion weights. A discrete Laplacian filter is applied to the grayscale version *G* = 0.299*R+0.587*G+0.114*B of the polar image to detect intensity discontinuities. Letting *G*′ denote the replicate-padded version of *G*, the contrast measure at pixel (*i, j*) is
  $$\begin{array}{cc} C\left(i,j\right)=| & G^{\prime }\left(i-1,j\right)+G^{\prime }\left(i,j-1\right)-4G^{\prime }\left(i,j\right)\\ & +G^{\prime }\left(i,j+1\right)+G^{\prime }\left(i+1,j\right)| \end{array}$$
  which corresponds to the absolute response of the Laplacian kernel
  $$F=\left[\begin{array}{ccc} 0 & 1 & 0\\ 1 & -4 & 1\\ 0 & 1 & 0 \end{array}\right].$$
  The pseudocode for the computing contrast is given in Algorithm 5.
2. Saturation: Saturation quantifies the vividness of color at each pixel, rewarding images that retain natural chromatic richness. Denoting the mean of the three color channels by
  $$A=\frac{R+G+B}{3},$$
  the saturation measure is defined as the standard deviation of the channel values about their mean:
  $$S=\sqrt{\frac{{\left(R-A\right)}^{2}+{\left(G-A\right)}^{2}+{\left(B-A\right)}^{2}}{3}}.$$
  The pseudocode for the computing saturation is given in Algorithm 6.
3. Well-exposedness: Pixels that are neither too dark nor too bright contribute most to a visually pleasing result. This is modeled by a Gaussian distribution centered at a target intensity *x*∈[0, 1] with standard deviation σ = 0.2, applied independently to each color channel:
  $$\begin{array}{ll} GR & =\exp\left(-\frac{{\left(R-x\right)}^{2}}{2\sigma^{2}}\right),\\ GG & =\exp\left(-\frac{{\left(G-x\right)}^{2}}{2\sigma^{2}}\right),\\ GB & =\exp\left(-\frac{{\left(B-x\right)}^{2}}{2\sigma^{2}}\right). \end{array}$$
  The per-pixel well-exposedness score is the product of the three channel responses:
  E=GR·GG·GB.
  For low-light images, setting *x* to a value slightly above 0.5 biases the fusion toward brighter polar images, further improving the overall brightness of the fused result. The pseudocode for the computing well-exposedness is given in Algorithm 7.

Algorithm 5. Contrast(*M*). 
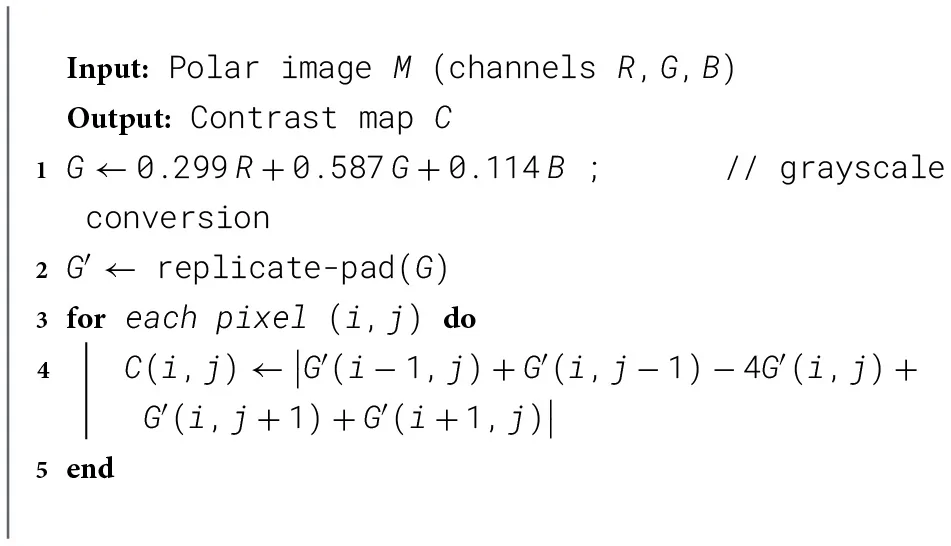

Algorithm 6. Saturation(*M*). 
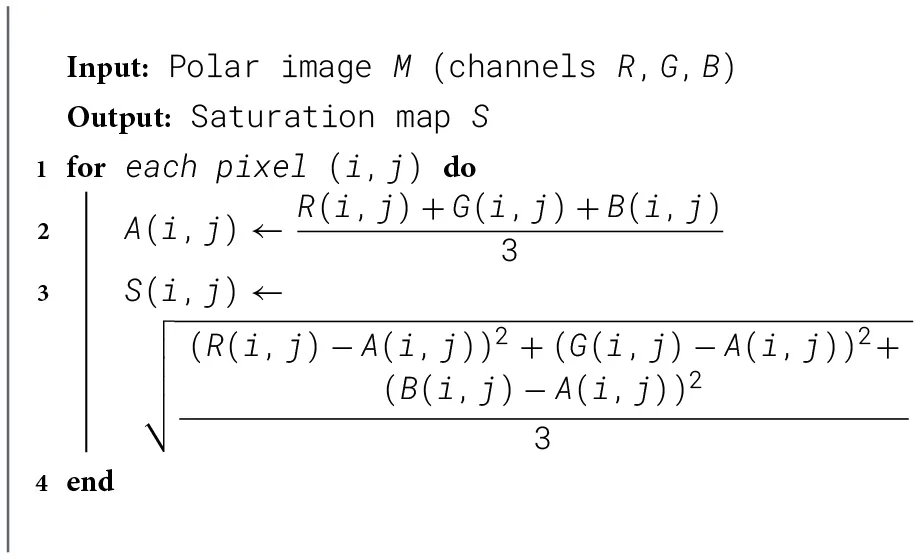

Algorithm 7. WellExposedness(*M, x*). 
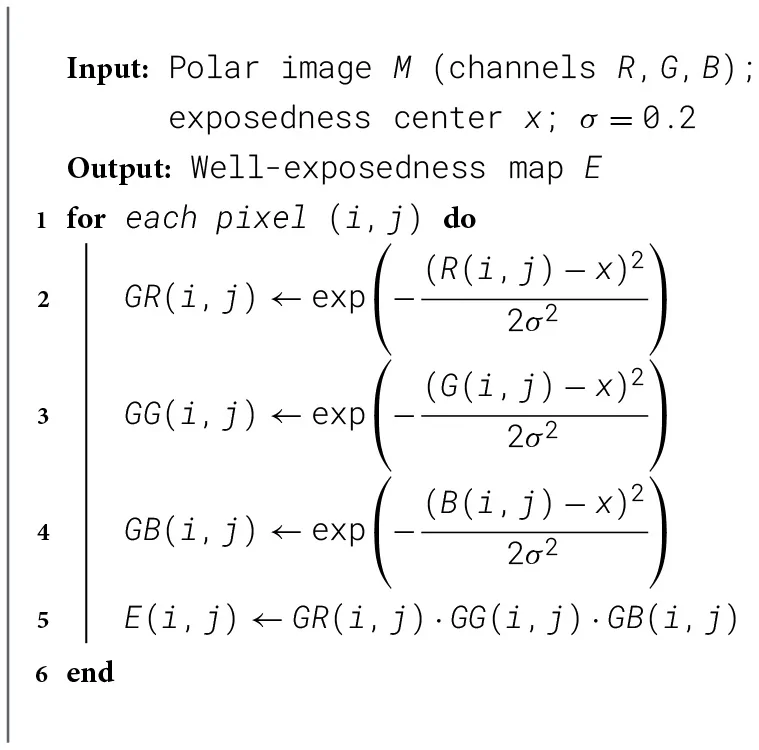

The combined weight map *W* is obtained as the weighted product of the three quality measures:

$$\begin{array}{l} W=C^{w_{c}}\cdot S^{w_{s}}\cdot E^{w_{e}}, \end{array}$$ (6)

where *w*_*c*_, *w*_*s*_, *w*_*e*_≥0 are user-specified exponents controlling the relative contribution of each measure. Setting any exponent to zero effectively removes the corresponding component from the weight map.

### 3.4 Fusion algorithm

Let {*M*_1_, *M*_2_, …, *M*_*m*_} denote the set of *m* polar images and {*W*_1_, *W*_2_, …, *W*_*m*_} their corresponding weight maps computed via Equation 4. To ensure a consistent contribution across images, the weight maps are normalized at each pixel location (*i, j*) so that they form a partition of unity:

$$\begin{array}{l} {\hat{W}}_{i,j,k}=\frac{W_{i,j,k}}{\overset{m}{\underset{q=1}{\sum }}W_{i,j,q}}, \end{array}$$ (7)

where *W*_*i, j, k*_ denotes the (*i, j*)-th entry of the weight map *W*_*k*_.

The fusion is carried out in the pyramid domain to avoid halo artifacts and gradient reversals that arise from direct blending in the spatial domain. From Figure 3 it can be clearly seen the pyramid fusion is far superior than the fusion carried in the spatial domain. Specifically, for each polar image *I*_*k*_, its Laplacian pyramid {*L*(*I*_*k*_)} is computed as described in Section 3.2. The Gaussian pyramid $\left\{G\left({\hat{W}}_{k}\right)\right\}$ of the corresponding normalized weight map is computed similarly. The fused pyramid levels are obtained as the weighted sum:

$$\begin{array}{l} R\left(F\right)=\overset{m}{\underset{k=1}{\sum }}L\left(I_{k}\right)\cdot G\left({\hat{W}}_{k}\right). \end{array}$$ (8)

Using the Gaussian pyramid of the weights (rather than the weights themselves) ensures that the blending transitions are spatially smooth at every scale. The final fused image *F* is then reconstructed from {*R*(*F*)} using the recursive procedure in Equation 3.

**Figure 3:**
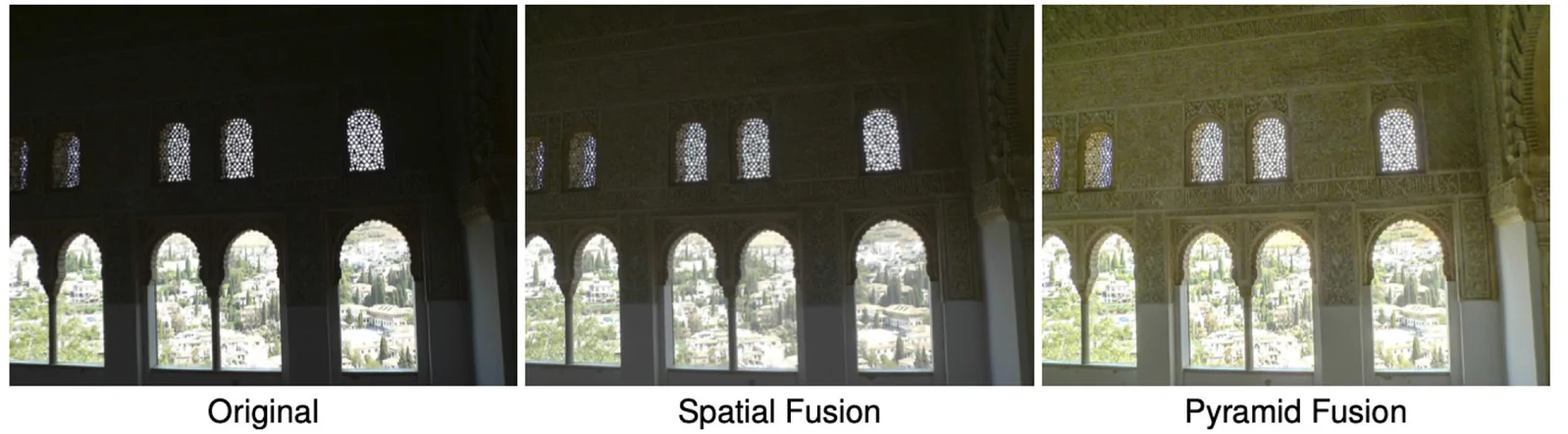
Comparison on spatial fusion (simple average) and pyramid fusion (image adapted with permission from The Most Difficult Image Enhancement Dataset [TM-DIED], Vasileios, V. [2025], https://sites.google.com/site/vonikakis/datasets/tm-died).

### 3.5 Defuzzification

After pyramid fusion, the fused image *F* resides in the normalized fuzzy domain [0, 1]. Defuzzification converts this continuous-valued representation back to the discrete 8-bit integer domain required for display and evaluation. The crisp output image *O* is obtained pixel-wise as:

$$\begin{array}{l} O_{mn}=\{\begin{array}{cc} 0, & \text{if}\;F_{mn}<0,\\ 255, & \text{if}\;F_{mn}>1,\\ \mathrm{round}\left(255F_{mn}\right), & \text{if}\;0\leq F_{mn}\leq 1, \end{array} \end{array}$$ (9)

where *O*_*mn*_ is the final integer-valued pixel at position (*m, n*). The clamping conditions (< 0 and >1) safeguard against numerical overflow or underflow that may arise during pyramid reconstruction, while the rounding operation maps the continuous membership value to the nearest valid gray level in {0, 1, …, 255}. The importance of linear defuzzifier is discussed in Rahman (2025).

## 4 Experimental analysis

All experiments were conducted on an Apple MacBook Pro equipped with an M2 chip featuring an 8-core CPU and an 8-core GPU, 8 GB of unified memory, 256 GB of SSD storage, and a memory bandwidth of 100 GB/s, running macOS Sequoia 15.3. The proposed method and all competing approaches were implemented and executed using MATLAB R2024a (The MathWorks, Inc., Natick, MA, USA) and Python 3.12.4 Python 3.12 (Python Software Foundation, Wilmington, DE, USA). The deep learning baselines were implemented using their publicly released code from the corresponding GitHub repositories, with all default settings retained. For the proposed method, the well-exposedness weight map uses *x* = 0.5, while the contrast, saturation, and exposedness weighting coefficients (*w*_*c*_, *w*_*s*_, and *w*_*e*_) are each set to 1.

### 4.1 Dataset description

Two publicly available benchmarks are employed in this study: the TM-DIED dataset (Vasileios, 2025) and the ExDark dataset (Yuen and Chee, 2019).

#### 4.1.1 TM-DIED

The TM-DIED dataset is widely regarded as one of the most demanding benchmarks for low-light image enhancement. It comprises 222 high-resolution images spanning indoor and outdoor scenes, many of which contain simultaneously underexposed and normally exposed regions within the same frame. The core challenge of this benchmark is to selectively brighten the dark regions while leaving the well-exposed areas visually unchanged. The images are captured under a wide variety of natural illumination conditions, including nighttime, sunset, overcast skies, and daylight, which further increases the difficulty of the enhancement task.

#### 4.1.2 ExDark

The ExDark dataset contains 7,363 low-light images distributed across 12 object categories: bicycle, boat, bottle, bus, car, cat, chair, cup, dog, motorbike, people, and table, with each category comprising between 503 and 801 images. The dataset encompasses diverse lighting conditions, ranging from dim ambient light and mixed low-and-normal illumination to near-total darkness and includes both indoor and predominantly outdoor scenes. Images are provided in both landscape and portrait orientations, with most resolutions not exceeding 1, 000 × 1, 000 pixels. For the purposes of this study, a representative subset of 337 images is drawn from the ExDark dataset.

### 4.2 Evaluation metrics and interpretation

#### 4.2.1 Metrics used

The following image quality metrics are employed to quantitatively evaluate the enhanced images:

- Structural Similarity Index Measure (SSIM): Assesses the perceived similarity between two images in terms of luminance, contrast, and structure, with values in [0, 1] where 1 indicates identical images.
- Absolute Mean Brightness Error (AMBE): Measures the absolute difference between the mean intensity of the enhanced image and that of the reference, quantifying the degree of brightness deviation introduced by the enhancement.
- Lightness Order Error (LOE): Evaluates the degree to which the relative lightness ordering among pixels is disturbed after enhancement; lower values indicate that the natural lightness ordering is better preserved.
- Lightness Order Measure (LOM): A complementary measure to LOE that quantifies how well the enhancement respects the perceptual lightness ordering of the original scene.
- Structure Measure Operator (SMO): Captures the extent to which structural information (edges and textures) is retained or distorted during enhancement.
- Blind/Referenceless Image Spatial Quality Evaluator (BRISQUE): An no-reference, block-based measure to estimate perceptual distortion through local variance and blockiness without a reference image, lower values indicate less visible artifacts and better perceptual quality.
- Entropy: Measures information content, randomness of an image according to its intensity distribution, high entropy usually indicates more details and textures while very high levels are often due to noise.
- Perception based Image Quality Evaluator (PIQE): No-reference, block based metric that estimates perceptual distortions through analysis of local variances and blocks within images, low values indicate less visible artifact.
- Fitness: Evaluates the overall fidelity of the enhanced image relative to a desired enhancement target (Eyob, 2023).
- Natural Image Quality Evaluator (NIQE): A no-reference metric that measures the statistical naturalness of the enhanced image by comparing its statistical features against a corpus of pristine natural images; lower NIQE scores correspond to perceptually higher quality.

#### 4.2.2 Interpreting metrics on real-world low-light images

An important distinction must be drawn between evaluation in deep-learning-based enhancement studies and evaluation on real-world low-light images. In supervised deep learning settings, ground-truth reference images are available, and metrics such as SSIM and PSNR directly measure fidelity to those references. A higher SSIM in that context unambiguously indicates better performance.

In the present study, however, ground-truth references are unavailable, and quality metrics are therefore computed by comparing the enhanced output against the original low-light input. Under this regime, metric values must be interpreted with care: a higher or lower score for a given metric does not straightforwardly imply superior or inferior enhancement. To illustrate, consider SSIM. If an enhancement method returns the input image entirely unchanged, the SSIM with respect to the input is exactly 1, the maximum possible value. This does not mean the method performs well; it means no enhancement has occurred. Conversely, a method that substantially brightens dark regions will inevitably alter pixel values, reducing SSIM relative to the dark input, yet the visual result may be far superior. The same reasoning applies to AMBE, LOE, and related reference-based metrics.

Accordingly, the numerical metric values reported in this study should be interpreted in conjunction with the corresponding visual results. Quantitative scores are used to characterize the behavior of each method, for instance, how much brightness is shifted or how well structure is preserved, rather than to rank methods by a single scalar threshold.

For example, Table 1 shows that SFCEF yields the highest AMBE value and LLM the lowest. As seen in Figure 4, this corresponds to over-enhancement and detail loss for SFCEF, and insufficient enhancement for LLM. The proposed method, however, preserves fine details while enhancing the image effectively, despite its AMBE value falling neither at the highest nor the lowest extreme.

**Table 1:** Quantitative evaluation of the proposed model under different *m*-polar configurations.

Module	SSIM	AMBE	LOE	LOM	SMO
[–0.3, 0, 1.2]	0.702 ± 0.1	1.768 ± 0.9	1.167 ± 0.9	0.113 ± 0.2	0.460 ± 0.6
[–0.3, 0, 1.4]	0.532 ± 0.2	2.992 ± 1.4	1.595 ± 1.1	0.210 ± 0.3	1.455 ± 2.0
[–0.3, 0, 1.5]	0.416 ± 0.2	4.571 ± 2.1	2.161 ± 1.3	0.337 ± 0.5	5.572 ± 7.9
[–0.6, 0, 1.2]	0.699 ± 0.1	1.708 ± 0.8	1.345 ± 1.0	0.129 ± 0.2	0.455 ± 0.6
[–0.6, 0, 1.4]	0.536 ± 0.2	2.852 ± 1.4	1.713 ± 1.2	0.214 ± 0.3	1.432 ± 2.0
[–0.6, 0, 1.5]	0.421 ± 0.2	4.396 ± 2.1	2.249 ± 1.4	0.328 ± 0.5	5.506 ± 7.9
[–0.75, 0, 1.2]	0.689 ± 0.1	1.836 ± 0.9	1.484 ± 1.0	0.152 ± 0.2	0.452 ± 0.6
[–0.75, 0, 1.4]	0.528 ± 0.2	2.965 ± 1.4	1.830 ± 1.2	0.232 ± 0.4	1.419 ± 2.0
[–0.75, 0, 1.5]	0.415 ± 0.2	4.491 ± 2.0	2.352 ± 1.4	0.338 ± 0.5	5.475 ± 7.8
[–0.6, –0.3, 0, 1.2, 1.4]	0.554 ± 0.2	2.686 ± 1.3	1.611 ± 1.1	0.190 ± 0.3	1.256 ± 1.7
[–0.75, –0.3, 0, 1.2, 1.5]	0.433 ± 0.2	4.090 ± 1.8	2.206 ± 1.2	0.274 ± 0.4	4.936 ± 7.3
[–0.75, –0.6, 0, 1.4, 1.5]	0.432 ± 0.2	4.091 ± 1.8	2.189 ± 1.4	0.292 ± 0.4	4.778 ± 7.0
[–0.75, –0.6, –0.3, 0, 1.2, 1.4, 1.5]	0.630 ± 0.2	3.642 ± 1.5	2.290 ± 1.0	0.556 ± 0.5	0.903 ± 2.3

Reported values are scaled for readability: AMBE values are multiplied by 10^−1^, LOE values by 10^−2^, and LOM values by 10^−4^.

**Figure 4:**
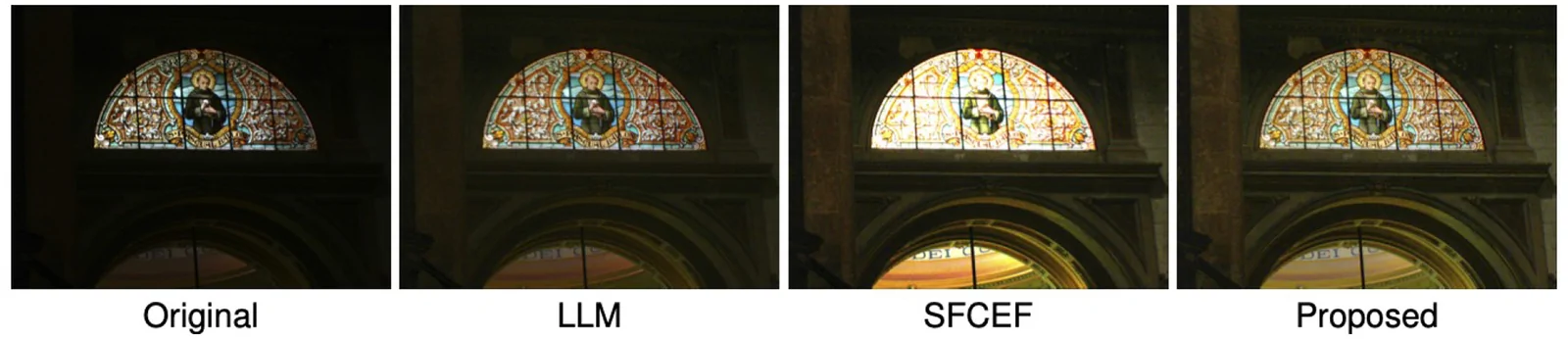
Comparison of a image generated using LLM, SFCEF and proposed (image adapted with permission from The Most Difficult Image Enhancement Dataset [TM-DIED], Vasileios, V. [2025], https://sites.google.com/site/vonikakis/datasets/tm-died).

### 4.3 Ablation study

#### 4.3.1 Effect of the number of polarities *m*

To investigate the contribution of the polarity count *m* and the choice of γ parameters, we evaluate the proposed framework across thirteen configurations spanning *m*∈{3, 5, 7}, as shown in Figure 5 and Tables 1, 2.

**Figure 5:**
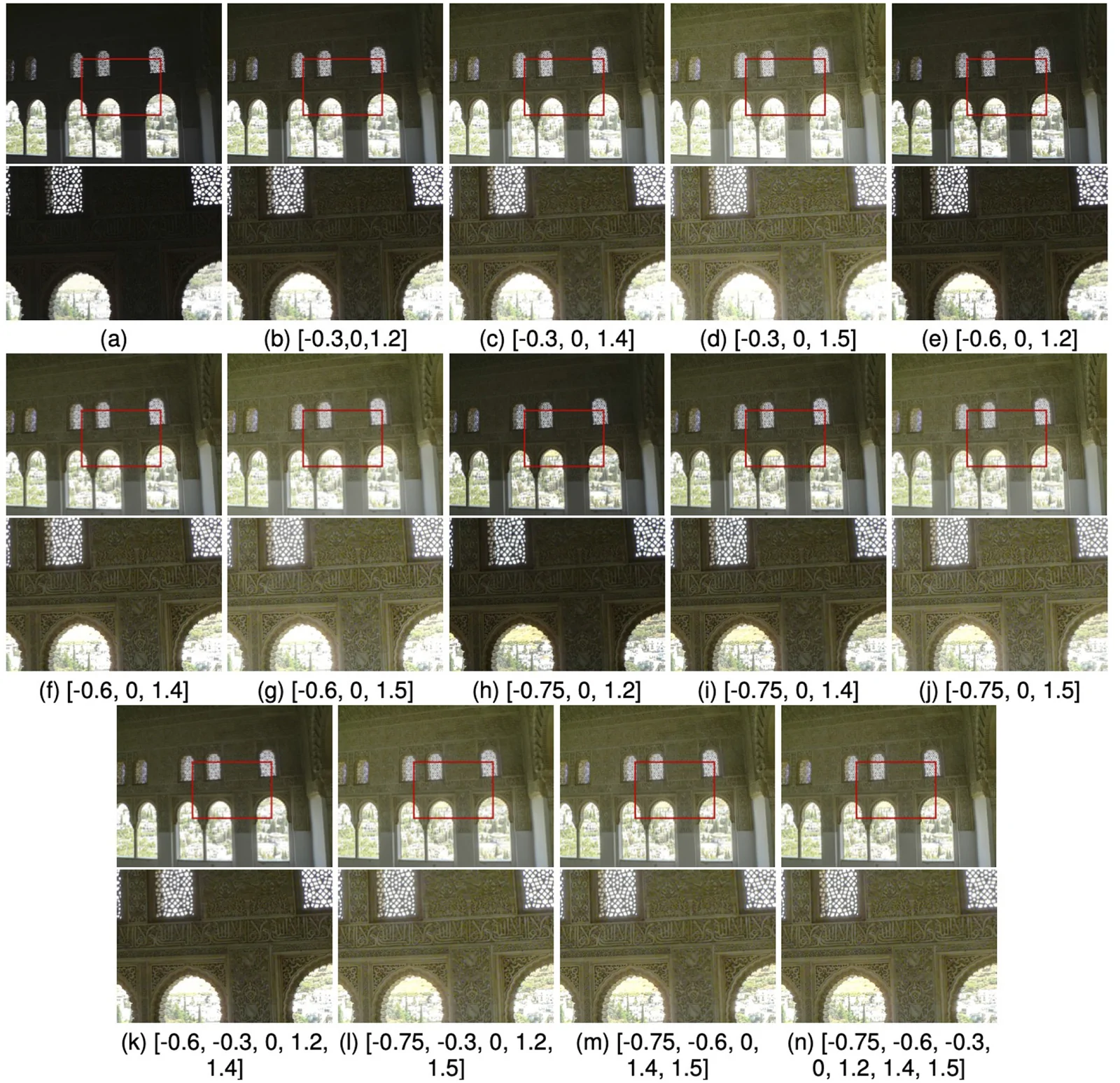
Ablation study on the effect of m in the m-polar framework (image adapted with permission from The Most Difficult Image Enhancement Dataset [TM-DIED], Vasileios, V. [2025], https://sites.google.com/site/vonikakis/datasets/tm-died). **(a)** Original input. **(b–j)** Results for *m* = 3 with varying γ combinations. **(k–m)** Results for *m* = 5 with varying γ combinations. **(n)** Result for *m* = 7.

**Table 2:** Quantitative evaluation of the proposed model under different *m*-polar configurations on no-reference metric.

Method	BRISQUE	Entropy	PIQE	Fitness	NIQE
[–0.3, 0, 1.2]	24.636 ± 8.7	7.250 ± 0.7	418.464 ± 8.5	0.151 ± 0.1	2.927 ± 1.0
[–0.3, 0, 1.4]	24.795 ± 8.9	7.344 ± 0.6	417.936 ± 8.0	0.174 ± 0.1	2.981 ± 1.1
[–0.3, 0, 1.5]	24.902 ± 9.7	7.337 ± 0.4	417.747 ± 7.5	0.195 ± 0.1	3.121 ± 1.2
[–0.6, 0, 1.2]	23.921 ± 9.6	7.187 ± 0.7	419.603 ± 8.6	0.153 ± 0.1	2.912 ± 1.0
[–0.6, 0, 1.4]	24.105 ± 9.2	7.332 ± 0.6	418.640 ± 8.0	0.176 ± 0.1	2.968 ± 1.1
[–0.6, 0, 1.5]	24.183 ± 9.8	7.376 ± 0.4	418.160 ± 7.5	0.197 ± 0.1	3.102 ± 1.2
[–0.75, 0, 1.2]	23.537 ± 9.8	7.165 ± 0.6	419.229 ± 8.5	0.155 ± 0.1	2.898 ± 1.0
[–0.75, 0, 1.4]	23.138 ± 9.8	7.340 ± 0.5	418.192 ± 7.8	0.177 ± 0.1	2.957 ± 1.1
[–0.75, 0, 1.5]	23.155 ± 10.1	7.408 ± 0.4	417.620 ± 7.2	0.198 ± 0.1	3.092 ± 1.2
[–0.6, –0.3, 0, 1.2, 1.4]	24.111 ± 9.2	7.328 ± 0.6	18.683 ± 8.0	0.173 ± 0.1	2.959 ± 1.1
[–0.75, –0.3, 0, 1.2, 1.5]	23.292 ± 10.1	7.440 ± 0.4	17.817 ± 7.3	0.197 ± 0.1	3.064 ± 1.2
[–0.75, –0.6, 0, 1.4, 1.5]	23.225 ± 10.3	7.421 ± 0.4	18.133 ± 7.4	0.197 ± 0.1	3.080 ± 1.2
[–0.75, –0.6, –0.3, 0, 1.2, 1.4, 1.5]	23.136 ± 10.2	7.430 ± 0.4	18.186 ± 7.4	0.204 ± 0.1	2.381 ± 0.6

The role of individual γ values can be understood intuitively: a negative γ (e.g., −0.75) produces a polar image that is darker than the normalized input, acting as a suppressor of over-enhancement in already well-lit regions. A positive γ (e.g., 1.5) yields a brighter polar image, amplifying low-intensity pixels. The identity polarity γ = 0 retains the original intensity distribution unchanged.

From the visual results in Figure 5, two observations stand out. First, configurations that exclude γ = 1.5 produce insufficiently bright outputs, indicating that a strongly positive polarity is necessary to recover detail in severely dark regions. Second, configurations that exclude γ = −0.75 tend to over-brighten normally exposed regions, confirming that at least one negative polarity is needed to suppress unwanted amplification in bright areas.

Comparing configurations with *m* = 7 ({−0.75, −0.6, −0.3, 0, 1.2, 1.4, 1.5}) against visually similar *m* = 3 ({−0.75, 0, 1.5}) and *m* = 5 variants ({−0.75, −0.3, 0, 1.2, 1.5}) and ({−0.75, −0.6, 0, 1.4, 1.5}), the perceptual differences appear minor. However, the quantitative results in Table 1 reveal that *m* = 7 achieves notably better structural preservation, as evidenced by the lowest NIQE score, higher SSIM and a competitive SMO value across all configurations. This improvement arises because denser sampling of the γ parameter space introduces intermediate polar images that preserve mid-tone structure more faithfully during fusion.

While increasing *m* raises computational cost proportionally (since each additional polarity requires a separate pyramid decomposition and weight map computation), the *m* = 7 configuration strikes the best balance between perceptual quality and structural fidelity. Accordingly, γ∈{−0.75, −0.6, −0.3, 0, 1.2, 1.4, 1.5} is adopted as the default configuration for all subsequent experiments.

#### 4.3.2 Effect of weight map components

Figures 6, 7 present a visual ablation of the three weight map components, contrast (*C*), saturation (*S*), and well-exposedness (*E*), evaluated on two representative images.

**Figure 6:**
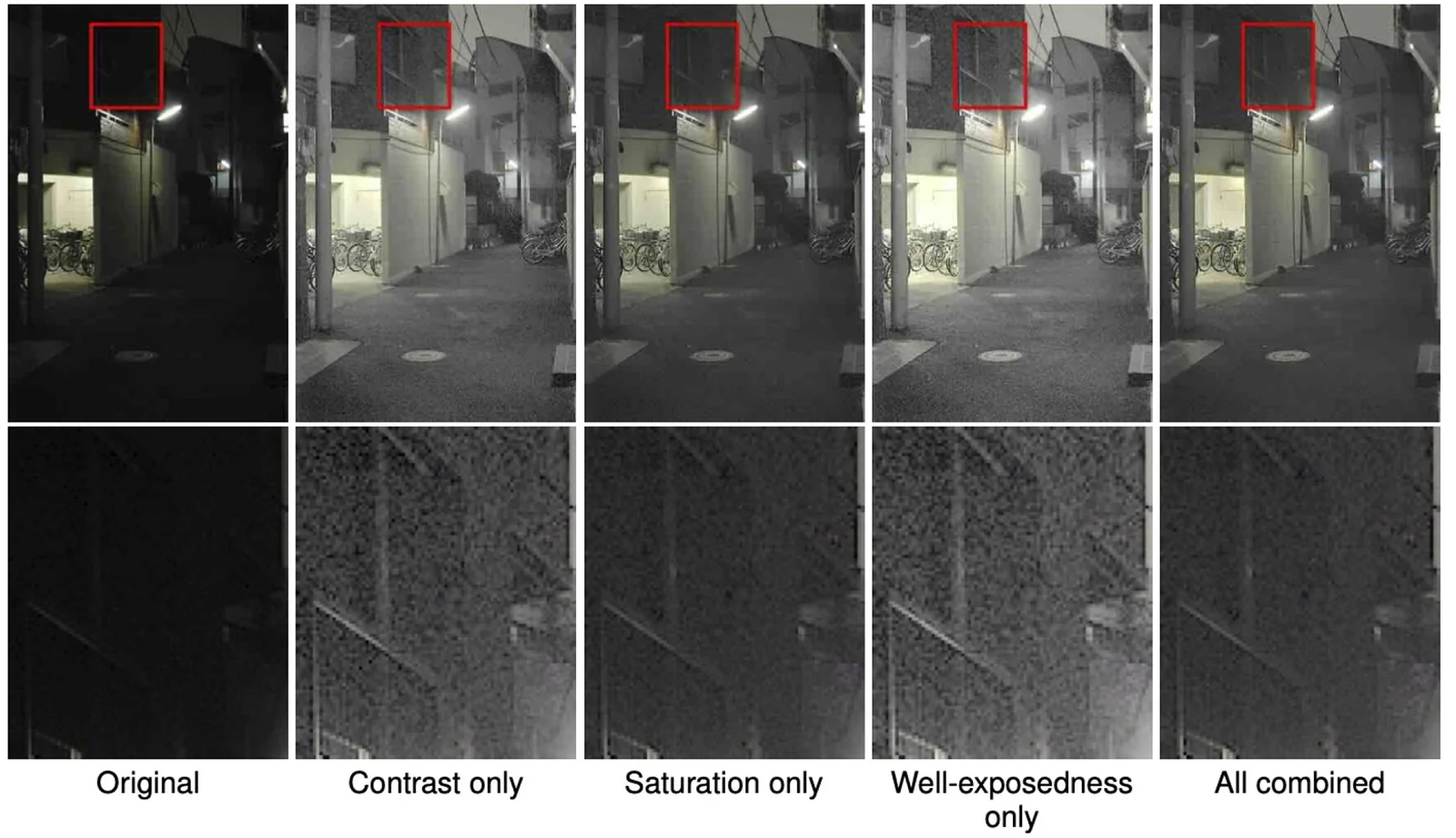
Ablation study on weight map components (images adapted with permission from the Exclusively-Dark-Image-Dataset [ExDark], Yuen, P. L., and Chee, S. C. [2019], https://github.com/cs-chan/Exclusively-Dark-Image-Dataset). Using contrast alone produces noise-amplified outputs; the saturation term suppresses this artefact.

**Figure 7:**
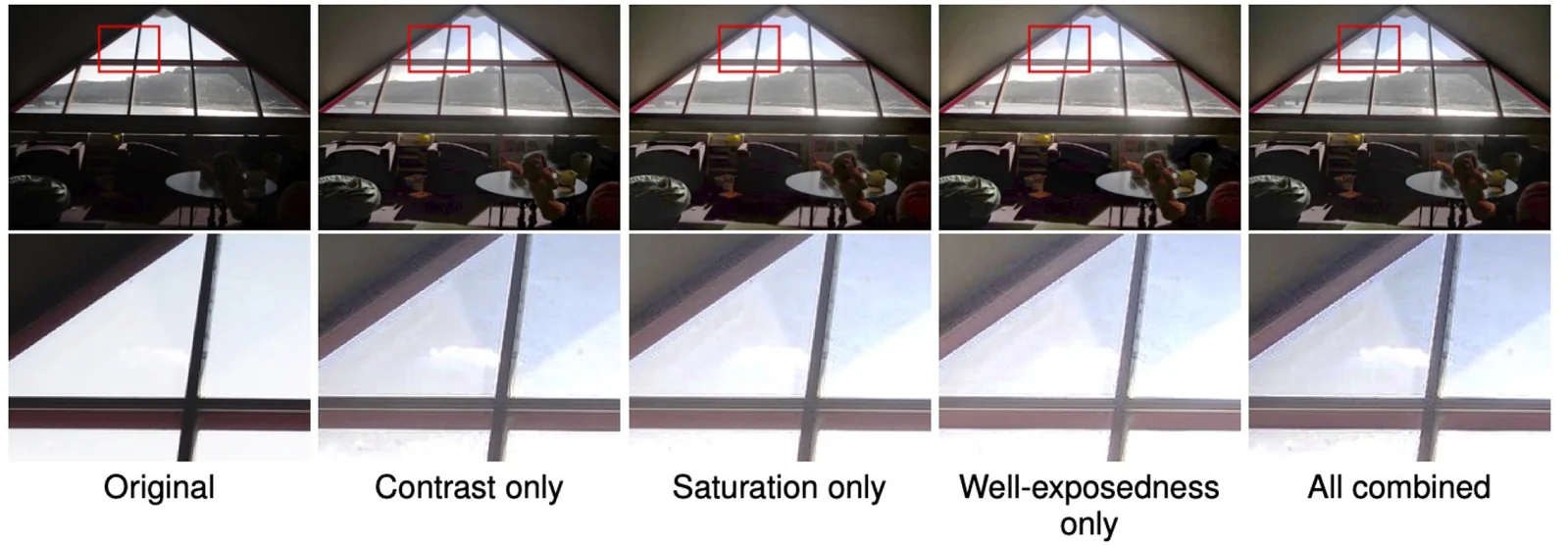
Ablation study on weight map components (images adapted with permission from the Exclusively-Dark-Image-Dataset [ExDark], Yuen, P. L., and Chee, S. C. [2019], https://github.com/cs-chan/Exclusively-Dark-Image-Dataset). Omitting the contrast term causes the loss of cloud boundary detail visible in the combined result.

Using only the contrast weight causes the fused image to exhibit pronounced noise amplification. This occurs because the Laplacian-based contrast measure assigns high weights to any region with strong local gradients, including noise-induced intensity fluctuations, without regard to color consistency or overall brightness. Similarly, relying solely on the well-exposedness weight introduces analogous artifacts, since the Gaussian exposure model assigns high weights purely based on proximity to a target intensity, without discriminating between genuine edges and noisy textures.

The saturation measure mitigates both of these failure modes by assigning higher weights to pixels that exhibit significant channel-to-channel variation, a property that is characteristic of genuinely colorful, noise-free regions rather than achromatic or noisy ones. As shown in Figure 6, the image fused using only saturation and well-exposedness lacks the sharp cloud boundaries visible in the combined result; these boundaries are recovered only when the contrast term is included, confirming that the Laplacian filter is responsible for preserving high-frequency edge structure.

The complementary nature of the three measures justifies their joint use. Each pairwise combination reveals a specific deficiency:

- *Contrast + well-exposedness only*: residual noise remains due to the absence of saturation-based color regularization.
- *Saturation + well-exposedness only*: edge detail is lost because no gradient-sensitive term is present.
- *Contrast + saturation only*: the fusion lacks guidance on how much each pixel should be brightened, as the well-exposedness term is the sole component that encodes target intensity information.

Only the full combination of all three measures yields an output that is simultaneously well-exposed, colorfully saturated, and edge-preserving.

### 4.4 Comparison with state-of-the-art methods

The proposed method is benchmarked against nine recent low-light image enhancement approaches: SCI (Long et al., 2022), URetinex-Net (Wenhui et al., 2022), EntropySDE (Lian et al., 2024), CSEC (Li et al., 2024), COLIE (Chobola et al., 2025), the parametrised intuitionistic fuzzy model for low-light image enhancement (PIFLOLIE) (Maheshkumar et al., 2025a), Reti-Diff (He et al., 2025), latent light manifestation (LLM) (Zohair, 2025), ALEN (Perez-Zarate et al., 2025), and SFCEF Rahman et al. (2025).

#### 4.4.1 Visual comparison

A distinguishing characteristic of many existing LLIE methods is that they are designed exclusively for images in which the entire scene is uniformly dark. In practice, however, low-light images frequently contain mixed-illumination regions, where dark areas coexist with normally exposed patches in the same frame. Enhancing such images without distorting the already well-lit regions is a substantially harder problem.

Figures 8, 9 illustrate this scenario on two representative mixed-illumination images from the TM-DIED dataset. SCI, URetinex-Net, EntropySDE, and Reti-Diff produce over enhanced outputs in both figures. COLIE introduces localized darkening near window boundaries, which degrades the perceptual quality of the enhanced image. PIFLOLIE achieves good detail recovery in the dark regions but, owing to its reliance on CLAHE, causes white structural elements (e.g., pillars and window frames) to appear darker than in the original, a well-known side effect of local histogram stretching. LLM and ALEN largely preserve the normally exposed regions and provide reasonable overall brightness, but fine structural detail in distant objects (e.g., tall buildings visible through a window) remains insufficiently recovered and lesser brightness compared to the proposed method. The proposed method enhances the dark regions effectively while preserving the normally exposed areas, and the cropped detail panels confirm its superiority in recovering fine structural content.

**Figure 8:**
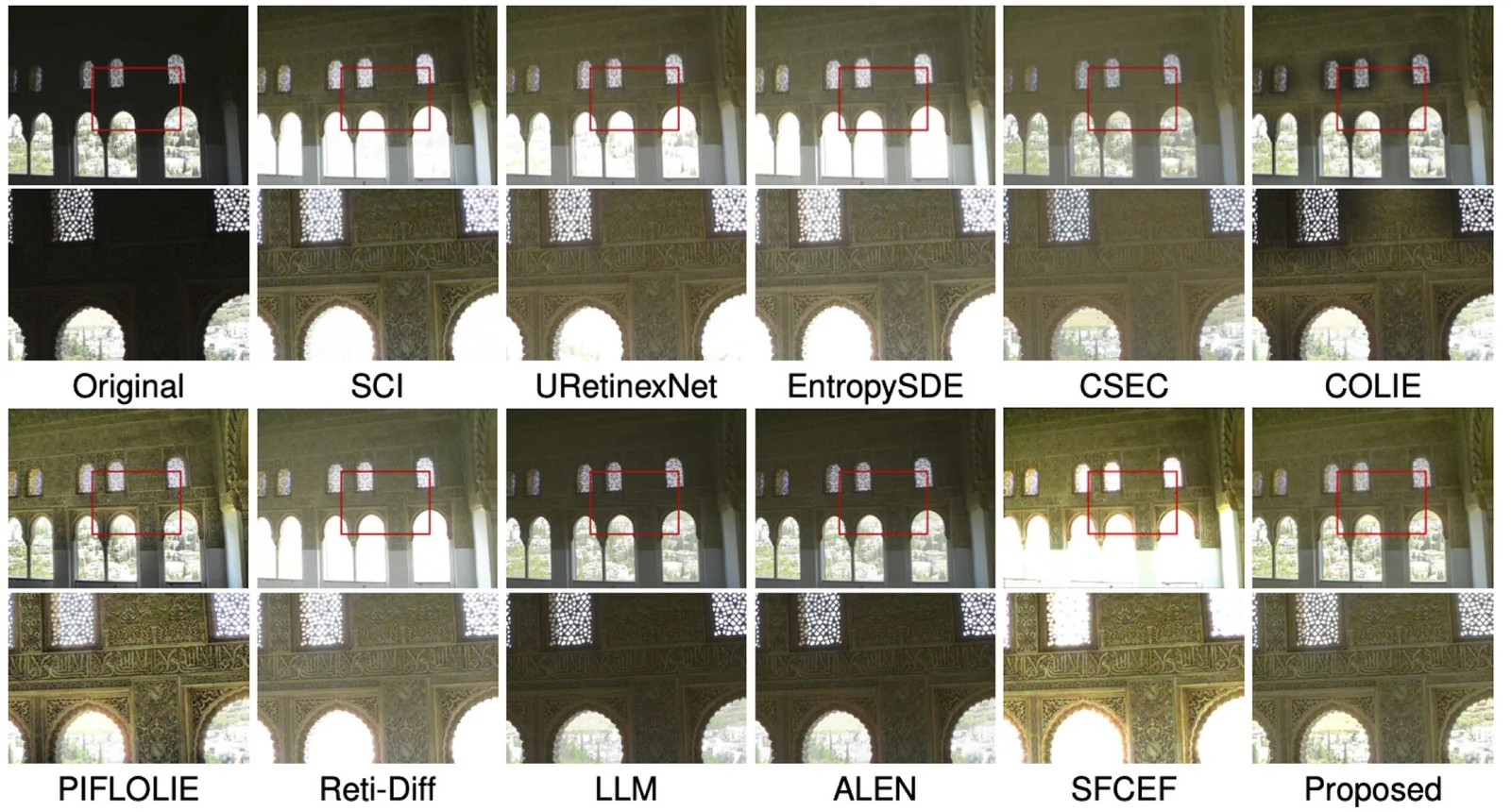
Visual comparison on a mixed-illumination image (input image adapted with permission from The Most Difficult Image Enhancement Dataset [TM-DIED], Vasileios, V. [2025], https://sites.google.com/site/vonikakis/datasets/tm-died). The red bounding box highlights the region of interest, which is magnified below each full image. The proposed method recovers fine structural detail in the dark region while leaving the normally exposed area undistorted.

**Figure 9:**
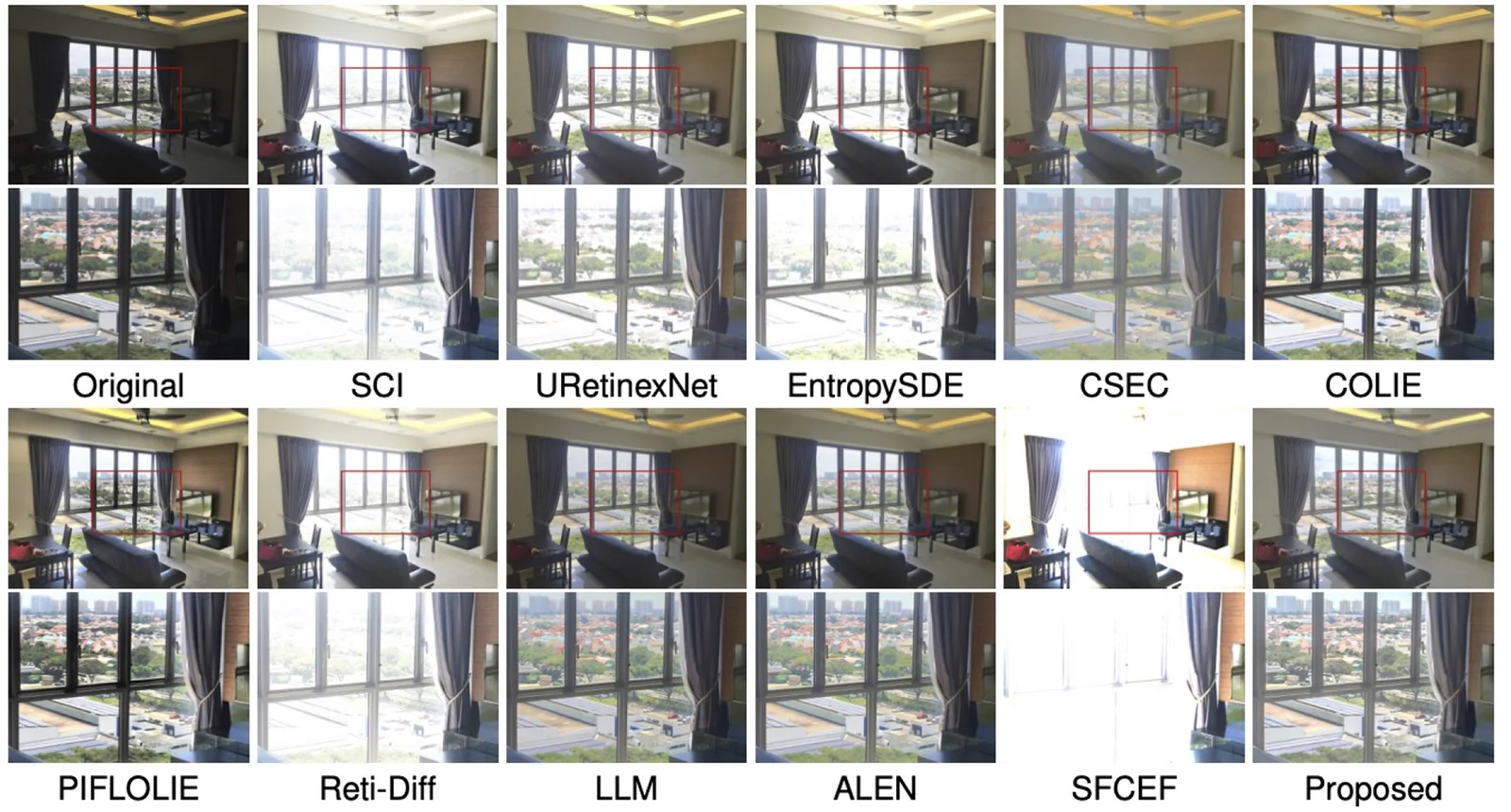
Visual comparison on a second mixed-illumination image (input image adapted with permission from The Most Difficult Image Enhancement Dataset [TM-DIED], Vasileios, V. [2025], https://sites.google.com/site/vonikakis/datasets/tm-died). The proposed method achieves balanced enhancement across both dark and normally exposed regions, outperforming competing methods in the magnified detail panels.

#### 4.4.2 Quantitative comparison

Tables 3, 4 report the quantitative results on the TM-DIED dataset with reference and no-reference metric, respectively. Tables 5, 6 report the quantitative results on the ExDark dataset with reference and no-reference metric, respectively As discussed in Section 4.2, all metrics are computed with respect to the original low-light input, so the direction of each metric must be interpreted contextually rather than by a fixed better-is-higher or better-is-lower rule.

**Table 3:** Quantitative comparison on the TM-DIED dataset on reference metrics.

Module	SSIM	AMBE	LOE	LOM	SMO
SCI	0.366 ± 0.1	7.793 ± 1.9	1.800 ± 1.4	1.981 ± 1.5	0.798 ± 1.2
URetinexNet	0.519 ± 0.2	5.656 ± 1.2	2.162 ± 1.1	1.336 ± 0.9	0.675 ± 2.3
EntropySDE	0.397 ± 0.1	7.223 ± 1.4	3.665 ± 1.9	4.665 ± 5.5	21.489 ± 52.9
CSEC	0.573 ± 0.2	4.894 ± 1.3	1.449 ± 0.7	0.760 ± 0.6	0.035 ± 0.1
COLIE	0.587 ± 0.1	4.837 ± 1.2	4.420 ± 1.7	0.727 ± 0.4	2.069 ± 6.7
PIFLOLIE	0.541 ± 0.2	4.506 ± 1.4	6.285 ± 3.3	1.593 ± 1.4	2.155 ± 5.1
Reti-Diff	0.407 ± 0.2	7.976 ± 1.8	3.323 ± 1.8	2.106 ± 1.7	0.087 ± 0.4
LLM	0.697 ± 0.1	2.840 ± 0.6	0.060 ± 0.1	0.642 ± 0.4	1.018 ± 2.8
ALEN	0.695 ± 0.1	3.001 ± 0.7	0.576 ± 0.6	0.556 ± 0.4	0.305 ± 0.5
SFCEF	0.227 ± 0.1	10.977 ± 2.6	9.939 ± 4.9	7.915 ± 5.9	0.890 ± 2.6
Proposed	0.630 ± 0.2	3.642 ± 1.5	2.290 ± 1.0	0.556 ± 0.5	0.903 ± 2.3

AMBE values are multiplied by 10^−1^, LOE values by 10^−2^, and LOM values by 10^−4^.

**Table 4:** Quantitative comparison on the TM-DIED dataset on no-reference metrics.

Method	BRISQUE	Entropy	PIQE	Fitness	NIQE
SCI	32.523 ± 8.2	6.257 ± 0.7	21.017 ± 7.8	0.154 ± 0.1	3.056 ± 1.3
URetinexNet	30.511 ± 8.7	6.862 ± 0.6	20.746 ± 8.4	0.169 ± 0.1	3.148 ± 1.4
EntropySDE	34.210 ± 8.7	6.224 ± 0.9	24.255 ± 9.8	0.146 ± 0.1	4.155 ± 2.3
CSEC	23.531 ± 8.9	7.358 ± 0.3	17.045 ± 8.4	0.172 ± 0.1	2.555 ± 0.5
COLIE	26.385 ± 8.8	7.358 ± 0.5	17.933 ± 7.6	0.171 ± 0.1	2.453 ± 0.6
PIFLOLIE	25.355 ± 10	7.738 ± 0.3	21.523 ± 6.7	0.220 ± 0.1	2.530 ± 0.6
Reti-Diff	31.292 ± 9.1	6.560 ± 0.9	31.106 ± 11.7	0.160 ± 0.1	3.571 ± 1.9
LLM	25.146 ± 8.9	7.181 ± 0.6	17.644 ± 8.4	0.158 ± 0.1	2.445 ± 0.5
ALEN	25.070 ± 9.9	7.325 ± 0.7	16.388 ± 8.1	0.169 ± 0.1	2.628 ± 0.5
SFCEF	44.965 ± 5.2	4.127 ± 1.3	41.712 ± 12.0	0.094 ± 0.0	6.892 ± 3.6
Proposed	23.136 ± 10.2	7.430 ± 0.4	18.186 ± 7.4	0.204 ± 0.1	2.381 ± 0.6

**Table 5:** Quantitative comparison on the ExDark dataset on reference metrics.

Module	SSIM	AMBE	LOE	LOM	SMO
SCI	0.380 ± 0.1	4.417 ± 2.3	0.566 ± 0.8	0.093 ± 0.1	3.840 ± 3.2
URetinexNet	0.289 ± 0.2	6.544 ± 1.9	2.152 ± 1.1	0.134 ± 0.1	1.657 ± 2.5
EntropySDE	0.211 ± 0.1	7.655 ± 2.1	3.078 ± 1.8	0.214 ± 0.2	114.732 ± 131.1
CSEC	0.264 ± 0.1	4.883 ± 2.1	1.731 ± 1.0	0.091 ± 0.1	0.202 ± 0.2
COLIE	0.363 ± 0.2	3.499 ± 1.3	2.882 ± 1.6	0.055 ± 0.1	14.574 ± 20.5
PIFLOLIE	0.252 ± 0.1	4.787 ± 1.7	4.311 ± 3.0	0.121 ± 0.1	12.146 ± 12.8
Reti-Diff	0.206 ± 0.1	7.057 ± 2.1	2.038 ± 1.2	0.187 ± 0.1	0.624 ± 1.9
LLM	0.509 ± 0.1	2.080 ± 0.8	0.011 ± 0.0	0.039 ± 0.0	6.133 ± 7.0
ALEN	0.463 ± 0.2	3.001 ± 1.9	1.515 ± 1.0	0.066 ± 0.1	0.905 ± 1.0
SFCEF	0.241 ± 0.2	6.979 ± 3.3	4.728 ± 3.6	0.354 ± 0.3	1.785 ± 4.7
Proposed	0.317 ± 0.2	4.110 ± 1.9	2.040 ± 1.4	0.078 ± 0.0	6.903 ± 7.5

AMBE values are multiplied by 10^−1^, LOE values by 10^−2^, and LOM values by 10^−4^.

**Table 6:** Quantitative comparison on the ExDark dataset on no-reference metrics.

Method	BRISQUE	Entropy	PIQE	Fitness	NIQE
SCI	32.962 ± 11.1	5.712 ± 1.3	41.884 ± 13.2	0.149 ± 0.1	3.816 ± 1.4
URetinexNet	28.687 ± 11.0	6.850 ± 1.0	42.735 ± 12.5	0.185 ± 0.1	3.325 ± 1.0
EntropySDE	26.560 ± 9.7	6.964 ± 0.8	40.845 ± 11.9	0.185 ± 0.1	3.283 ± 1.0
CSEC	30.632 ± 12.4	6.814 ± 0.8	39.000 ± 13.5	0.163 ± 0.1	3.106 ± 1.0
COLIE	32.650 ± 11.7	6.339 ± 1.1	39.726 ± 13.1	0.154 ± 0.1	3.570 ± 1.2
PIFLOLIE	34.138 ± 11.2	6.602 ± 1.0	42.317 ± 12.2	0.196 ± 0.1	3.985 ± 1.4
Reti-Diff	27.480 ± 10.9	7.061 ± 0.8	47.588 ± 12.4	0.191 ± 0.1	3.481 ± 1.0
LLM	31.970 ± 12.4	5.545 ± 1.3	39.065 ± 13.9	0.123 ± 0.1	3.532 ± 1.2
ALEN	34.064 ± 11.7	6.012 ± 1.3	41.582 ± 13.2	0.152 ± 0.1	3.506 ± 1.2
SFCEF	43.750 ± 8.4	5.588 ± 1.3	50.780 ± 15.7	0.125 ± 0.1	4.316 ± 1.3
Proposed	29.524 ± 11.2	6.679 ± 0.9	40.075 ± 12.6	0.190 ± 0.1	3.513 ± 1.2

On the TM-DIED dataset (Table 3), EntropySDE achieves the lowest SSIM, indicating that its output deviates substantially from the original input, a consequence of aggressive over-enhancement of the normally exposed regions. Reti-Diff shows a similar trend on ExDark (Table 5). LLM attains the highest SSIM on both datasets, closely followed by ALEN and the proposed method; this reflects the fact that all three methods avoid over-brightening the normally exposed regions. The proposed method scores slightly lower in SSIM than LLM on ExDark, which is attributable to its more aggressive brightening of the genuinely dark regions, a desirable characteristic that is obscured by the input-referenced metric. The LOE metric quantifies the extent to which the original brightness ordering among pixels is disturbed by enhancement. LLM achieves the lowest LOE on both datasets, suggesting that its output is closest in brightness distribution to the original low-light input. While a lower LOE is generally desirable, a very low LOE combined with a moderate AMBE, as observed for LLM, can indicate insufficient enhancement of the dark regions. LOM captures the degree of over-enhancement; the high LOM of EntropySDE confirms its tendency to over-amplify pixel intensities. SMO reflects structural divergence from the original image: the exceptionally high SMO of EntropySDE and COLIE on ExDark indicates severe structural distortion. LLM, ALEN, and the proposed method all exhibit comparatively low SMO values, consistent with their visual behavior of preserving normally exposed regions.

Regarding blind spatial quality, the proposed method achieves the lowest BRISQUE score among all methods on TM-DIED (23.136), indicating the fewest distortions. On ExDark, the proposed BRISQUE score (29.524) is higher than EntropySDE and Reti-Diff, but still lower than the remaining methods. In Entropy, the proposed method attains one of the highest values on TM-DIED (7.430), trailing only PIFLOLIE and indicating that fine detail and texture are well preserved. On ExDark, the proposed Entropy score (6.679) is moderately lower than Reti-Diff and EntropySDE, but remains higher than COLIE, PIFLOLIE, ALEN, SCI, LLM, and SFCEF. With respect to PIQE, the proposed method records a value of 18.186 on TM-DIED, closely trailing the lowest-scoring methods, ALEN and CSEC, and indicating relatively few visible blocking artifacts. On ExDark, the proposed method score (40.075) similarly remains close to the best-performing methods, CSEC (39.000) and LLM (39.065), and is lower than the other approaches.

Spider plots are used to visualize and compare performance across multiple metrics simultaneously, making trade-offs and the relative balance of each method's performance immediately apparent in a way that tabulated values alone cannot convey. Figure 10 depicts the spider plot of the proposed method with the state of the art methods comparing the quantitative metrics. Table 7 reports the total processing time of the method over the entire datasets. The higher computational cost relative to SCI, LLM and SFCEF is attributable to the multi-scale pyramid decomposition performed independently for each of the seven polar images, representing a trade-off accepted in favor of the structural quality gains demonstrated above. The proposed method takes only 0.495G Flops for an input image of size 512 × 512 × 3, and takes only *O*(*N*) complexity where *N* is the number of pixels.

**Figure 10:**
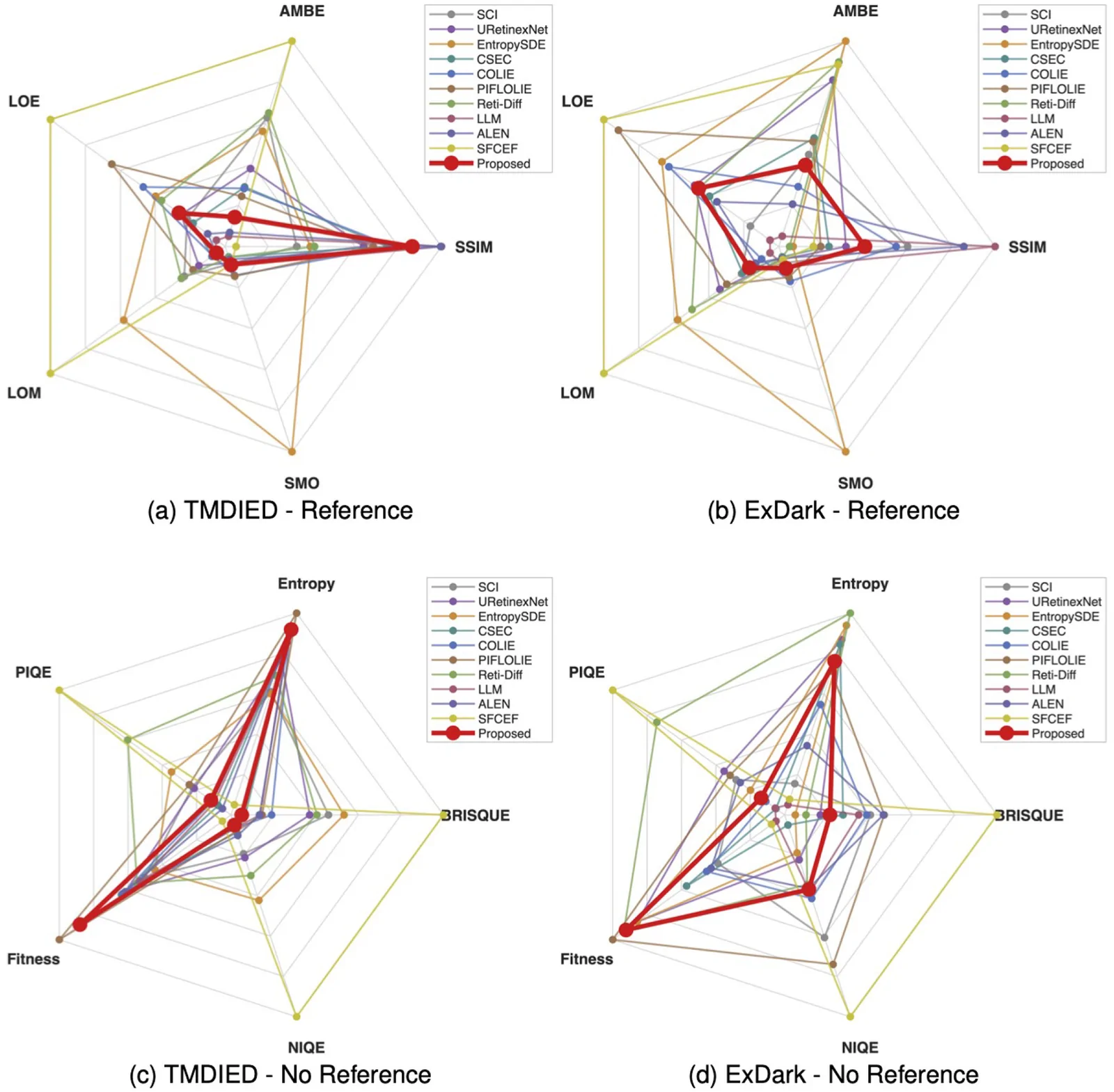
Spider plots comparing all state-of-the-art methods across quantitative reference and no-reference metrics on the TM-DIED and ExDark datasets. **(a)** TM-DIED, reference metrics **(b)** ExDark, reference metrics (SSIM, AMBE, LOE, LOM, SMO); **(c)** TM-DIED, no-reference metrics **(d)** ExDark, no-reference metrics (BRISQUE, Entropy, PIQE, Fitness, NIQE). Each axis is min–max normalized across methods for visual comparability. The proposed method is highlighted in red.

**Table 7:** Runtime comparison of all methods. Times are reported as total processing time (in seconds) over the respective test set.

Method	SCI	URetinexNet	EntropySDE	COLIE	PIFLOLIE	Reti-Diff	LLM	CSEC	ALEN	SFCEF	Proposed
Runtime (seconds)	451	2,816	34,253	1,268	2,610	147,165	933	9,998	25,038	279	9,087

### 4.5 Limitation and future direction

#### 4.5.1 Limitations

1. The reliance on the original image as a reference for full-reference metric computation, due to the unavailability of non-uniformly illuminated image datasets with ground-truth.
2. Although one of the aim of this study was to propose a simple LLIE method, the proposed approach is, somewhat complicated, because of the additional processing requirement to achieve balanced performance across metrics.
3. The model is not suitable if the low light image contains the over exposed region. The proposed model does not reduce the enhancement in the over exposed region it retains them.

#### 4.5.2 Future direction

1. Developing a non-uniformly illuminated image dataset with true ground-truth, enabling rigorous full-reference evaluation of illumination correction methods.
2. Designing a simpler multi-scale pyramid decomposition and incorporating it into the pipeline to reduce complexity while preserving enhancement quality.
3. Investigating image-adaptive parameter selection strategies and extending the method to video sequences.

## 5 Conclusion

This article proposed an *m*-polar fuzzy set-based framework for low-light image enhancement, in which *m* polar images are constructed via parametric involutive fuzzy complement functions and fused through a multi-scale Laplacian pyramid guided by contrast, saturation, and well-exposedness weight maps. The framework addresses the practically important but widely overlooked problem of mixed-illumination enhancement, where negative-γ polarities suppress over-brightening in normally exposed regions while positive-γ polarities recover detail in dark areas. Ablation studies confirmed the necessity of all three weight map components and justified the adopted *m* = 7 configuration. Experimental evaluation on the TM-DIED and ExDark datasets against nine state-of-the-art methods demonstrated that the proposed method achieves competitive performance across all metrics, without requiring any training data or paired ground-truth references.

## Data Availability

The original contributions presented in the study are included in the article/supplementary material, further inquiries can be directed to the corresponding author.
